# Association Between Oral Health-Related Quality of Life and Psychological Health Indicators in Older Adults

**DOI:** 10.1192/j.eurpsy.2026.11810

**Published:** 2026-07-31

**Authors:** C. Tsironis, S. Mantzoukas, A. Nakou, E. Bartzou, E. Dragioti, M. Gouva

**Affiliations:** 1 Scientific Laboratory of Psychology and Person-Centered Care; 2 Nursing, University of Ioannina, Ioannina, Greece

## Abstract

**Introduction:**

Oral health in older adults is a critical yet often underestimated determinant of well-being. Beyond chewing and nutrition, oral health reflects psychological functioning, daily activities, and overall quality of life. The *Geriatric Oral Health Assessment Index* (GOHAI) is widely used to assess oral health-related quality of life in elderly populations.

**Objectives:**

The aim of the present study was to investigate the relationship between oral health, as measured by the GOHAI, and indicators of physical and mental health in a sample of older adults in Greece.

**Methods:**

A total of 608 older adults (aged 65–99 years) participated in the study. Participants completed a questionnaire including demographic data, the GOHAI, the SF-36 Health Survey, and items regarding dentures and dental bridges. Descriptive statistics, Pearson’s correlations, and group comparisons (t-tests/ANOVAs) were conducted to explore associations between variables.

**Results:**

Analyses revealed that GOHAI scores were significantly and positively correlated with the SF-36 Physical Health component (*r* ≈ 0.42, *p* < 0.001), indicating that better oral health-related quality of life was associated with higher physical well-being. Moreover, group comparisons showed differences in GOHAI scores according to the presence of dentures or bridges, with participants without prosthetic restorations reporting more favorable outcomes.

**Conclusions:**

These findings highlight the importance of oral health as a key factor influencing the quality of life in older adults. Oral health should not be considered in isolation but integrated into comprehensive health promotion programs. Interdisciplinary collaboration among dentists, physicians, and psychologists is essential to improve elderly well-being, emphasizing interventions that combine both physical and psychological aspects of care.

**Disclosure of Interest:**

None Declared

